# Antiviral Potential of Green-Synthesized Zinc Oxide and Copper Oxide Nanoparticles: Integrated In Vitro and In Silico Study

**DOI:** 10.3390/nano16171067

**Published:** 2026-08-27

**Authors:** Atef S. Elgebaly, Yasmin Adel Elmahdy, Mujtaba Farooq Rana, Reem Mohammed Abdelrahman, Marwa M. Khalifa, Ehab Darwish, Ayman K. Ismail, Khamael M. Abualnaja, Mohamed Shaalan, Ahmed Mahdy

**Affiliations:** 1 Pharmacognosy Department, Faculty of Pharmacy, Ahram Canadian University, Giza 12573, Egypt; atefsamy.elgebaly@acu.edu.eg; 2 Central Laboratory Department, Memorial Souad Kafafi Hospital, Misr University of Science and Technology (MUST), Giza 12568, Egypt; 3 Clinical and Chemical Pathology Department, Faculty of Medicine, Cairo University, Cairo 11956, Egypt; dryasminadel1983@gmail.com; 4 Department of Biochemistry, College of Medicine, Taif University, Taif 26571, Saudi Arabia; 5 Microbiology and Immunology Department, Faculty of Medicine, Helwan University, Cairo 11795, Egypt; reem.abdelrahman@med.helwan.edu.eg; 6 Department of Microbiology and Immunology, Faculty of Pharmacy, Assiut University, Assiut 71526, Egypt; marwamohammed@pharm.aun.edu.eg; 7 Internal Medicine Department, College of Medicine, King Faisal University, Al-Ahsa 36362, Saudi Arabia; 8 Department of Forensic Medicine and Toxicology, College of Veterinary Medicine, Suez Canal University, Ismailia 41522, Egypt; 9 Department of Clinical Pharmacology and Forensic Medicine and Toxicology, College of Medicine, Taif University, Taif 26571, Saudi Arabia; 10 Department of Chemistry, College of Science, Taif University, Taif 21944, Saudi Arabia; 11 Department of Pathology, Faculty of Veterinary Medicine, Cairo University, Giza 12211, Egypt; 12 Molecular Biology and Biotechnology Department, School of Biotechnology, Badr University in Cairo (BUC), Cairo 11829, Egypt

**Keywords:** green synthesis, zinc oxide nanoparticles (ZnO-NPs), copper oxide nanoparticles (CuO-NPs), antiviral activity, HPV (human papillomavirus), rotavirus (ROTA), cytomegalovirus (CMV), MD simulation, Boltz-2, docking

## Abstract

Viral infections such as human papillomavirus, rotavirus, and cytomegalovirus continue to pose significant global health challenges, causing persistent and widespread disease. The objective of this study is to evaluate the preliminary in vitro antiviral and cytotoxicity profile of zinc oxide (ZnO) and copper oxide (CuO) nanoparticles, which were synthesized through a green approach using *Moringa oleifera* leaf extract, against these viruses. Regarding antiviral effects, ZnO nanoparticles demonstrated potent antiviral activity against HPV, CMV, and rotavirus, with high selectivity indices and lower cytotoxicity compared with copper oxide nanoparticles. For HPV and CMV, ZnO showed markedly higher CC_50_ and SI values (HPV: SI = 10.67; CMV: SI = 10.57) than copper oxide nanoparticles, which exhibited lower selectivity and greater cytotoxicity. Against rotavirus, ZnO nanoparticles also exhibited superior safety and antiviral efficacy (CC_50_ = 798.42 µg/mL; SI = 11.49), whereas copper oxide nanoparticles showed relatively higher toxicity and lower selectivity. In parallel, quercetin, the major flavonoid of *Moringa oleifera*, was evaluated in silico as a potential inhibitor of rotavirus VP5*, CMV UL44, and HPV E6 using molecular docking and 150 ns molecular dynamics simulations. Quercetin exhibited favorable binding affinity, stable interaction profiles, and low RMSD fluctuations across all viral targets. Overall, green-synthesized ZnO nanoparticles demonstrated promising preliminary antiviral activity with an improved safety profile compared with CuO nanoparticles, while the computational findings support quercetin as a potential multi-target antiviral candidate. Further studies using direct virological assays and in vivo models are required to validate these findings and elucidate the underlying antiviral mechanisms.

## 1. Introduction

Viral diseases remain a major global health challenge, particularly those caused by widespread and persistent viruses such as human papillomavirus (HPV), rotavirus (ROTA), and cytomegalovirus (CMV) [1]. HPV is the most common sexually transmitted virus and is directly linked to cervical cancer as well as other anogenital and oropharyngeal cancers [2]. Rotavirus is a leading cause of dehydrating gastroenteritis in infants and young children worldwide, with significant morbidity and mortality, especially in developing countries [3]. Cytomegalovirus (CMV), a ubiquitous member of the Herpesviridae family, establishes lifelong persistence and poses serious risks for immunocompromised subjects, transplant recipients, and congenitally infected neonates [4]. The limited effectiveness of current antiviral therapies and the rise in drug-resistant strains create an urgent need for new antiviral strategies [5]. Nanotechnology has become a promising approach in antiviral research, especially for infections such as COVID-19, by using nanomaterials with unique properties that boost antiviral activity [6]. Copper and zinc nanoparticles have gained attention for their strong antiviral potential, affordable production, and biocompatibility [7]. Copper oxide (CuO) nanoparticles generate reactive oxygen species (ROS) and alter viral proteins and genetic material, while zinc nanoparticles interrupt viral replication, protein synthesis, and host cell entry [8].

There has been growing interest in environmentally friendly synthesis methods for metal nanoparticles, using plant extracts, microorganisms, or natural biomolecules as reducing and stabilizing agents in place of hazardous chemicals and extreme reaction conditions [9,10,11]. This green chemistry approach offers several advantages over conventional physical and chemical synthesis, including lower energy consumption, reduced use of hazardous reagents, higher biocompatibility, and cost-effective scalability [10,11]. The biochemicals present in natural extracts can additionally act synergistically to stabilize nanoparticles and contribute to their antiviral efficacy [12]. Copper and zinc NPs prepared through such green routes have shown potent antiviral activity against HPV, rotavirus, and CMV by disrupting viral structures, preventing attachment, or interfering with replication [13]. Nanoparticle production using *Moringa oleifera* in particular offers improved biocompatibility and lower toxicity relative to traditionally synthesized nanomaterials [14], contributing to the development of sustainable antiviral nanomaterials with biomedical relevance [15].

The computational study focused on flavonoids identified in *Moringa oleifera* extract, prioritizing quercetin because it is reported as the most abundant flavonoid in this plant (2030.9 µmol/100 g) compared to other constituents such as chlorogenic acid (5.084 µmol/100 g), rutin (91.081 µmol/100 g) and kaempferol (69.09 µmol/100 g) [16]. quercetin was selected as the representative compound also because of its ability to chelate transition metal ions such as copper and zinc through its 5-OH and 4-carbonyl groups, as demonstrated by mass spectrometric studies and recent reviews on metallo-flavonoids [16] and belongs to the same phytochemical class implicated by the FTIR-detected capping/stabilizing functional groups on both ZnO-NPs and CuO-NPs (Section 3.1 and Section 3.2), providing a direct compositional link between the nanoparticle surface chemistry and the antiviral screening target. Flavonoids as a class are also recognized as multi-target ligands capable of engaging structurally distinct proteins [17], motivating docking and MD evaluation of Quercetin against three mechanistically unrelated viral targets (VP5*, UL44, E6) alongside three comparator flavonoids (chlorogenic acid, kaempferol, rutin). Rotavirus VP5* is a spike protein of the viral entry apparatus [18]; CMV UL44 is the processivity factor of the viral DNA polymerase and is essential for the replication cycle [19]; and HPV E6 oncoprotein inactivates the tumour suppressor p53 [20].

A comprehensive computational study predicted ligand–protein binding affinity using a multimodal co-folding model (Boltz-2) [21], while another study investigated the dynamical stability of protein–ligand complexes through molecular dynamics simulations using GROMACS 2024.2 [22].

The present study addresses this gap by directly comparing the antiviral efficacy and cytotoxicity profiles of green-synthesized ZnO and CuO nanoparticles across three virologically distinct pathogens (HPV, rotavirus, and CMV) within a single experimental framework, and by coupling these in vitro findings with an in silico investigation of quercetin, the major flavonoid of the same *Moringa oleifera* extract used for nanoparticle synthesis, against three mechanistically distinct viral targets. The novelty of this work therefore lies not in any single assay but in the integration of comparative multi-virus nanoparticle screening with a unified computational assessment of the phytochemical mediator common to both nanomaterials, offering a preliminary, extract-linked rationale for antiviral activity. Given the in vitro and in silico nature of the present study, the findings should be interpreted as an initial screening platform requiring further mechanistic and preclinical validation.

## 2. Materials and Methods

### 2.1. Preparation of Moringa oleifera Leaf Extract

Preparation of *Moringa oleifera* leaf extract: Fresh *Moringa oleifera* leaves were washed sequentially with running tap water and distilled water to remove surface debris, then air-dried and powdered. Ten grams of the dried leaf powder were suspended in 100 mL of distilled water (10% *w*/*v*) and heated under constant stirring (500 rpm, 90 °C) for 15 min. The mixture was filtered through Whatman No. 1 filter paper to obtain a clear aqueous extract and centrifuged at 2400 rpm for 5 min to remove debris. The supernatant was collected for nanoparticle synthesis. When larger extract volumes were required, the same extraction procedure was repeated using the identical powder-to-water ratio (10% *w*/*v*) to obtain the required volume of extract.

### 2.2. Synthesis of Zinc Oxide (ZnO) Nanoparticles

To prepare ZnO nanoparticles, 4.5 g of zinc acetate dihydrate, Zn (CH_3_CO_2_)2·2H_2_O (Sigma-Aldrich, St. Louis, MO, USA) 99.99% purity; ≈0.205 M in the 100 mL precursor solution), was dissolved in 100 mL of distilled water. *Moringa oleifera* leaf extract was added dropwise to the zinc acetate solution at an extract-to-precursor ratio of 5:1 (*v*/*v*) (100 mL extract:20 mL precursor), under continuous stirring for approximately 3 min, while heating at 70 °C for 2 h [23]. The mixture was subsequently maintained at room temperature for 2 h before nanoparticle recovery. An evident color change indicated the formation of ZnO nanoparticles. To remove impurities, the precipitate obtained was centrifuged at 5000 rpm for 30 min, followed by washing with distilled water and ethanol. The product was dried in an oven (200 °C, 2 h) and calcined at 400 °C for 2 h to obtain ZnO nanoparticles [23].

### 2.3. Synthesis of Copper Oxide (CuO) Nanoparticles

For CuO nanoparticle synthesis, the aqueous *Moringa oleifera* extract was pipetted into a 10 mM copper sulfate (CuSO_4_·5H_2_O) solution at an extract-to-precursor ratio of 4:1 (*v*/*v*) (320 mL extract:80 mL precursor), and the mixture was kept under continuous stirring at 700 rpm at 80 °C for 24 h. The pH of the reaction mixture was adjusted to 10.5 using 1 M NaOH. Formation of (CuO-NPs) was visually confirmed by a color change from light green to reddish brown. The nanoparticles were harvested by centrifugation at 5000 rpm for 30 min, washed with deionized water [24]. The extended drying period at 60 °C may facilitate partial surface oxidation of metallic copper. Therefore, the crystalline phase of the synthesized material was confirmed by XRD analysis, which demonstrated that the final product predominantly consisted of crystalline CuO nanoparticles.

### 2.4. Characterization of Nanoparticles

The formation of ZnO and CuO nanoparticles was evaluated using physicochemical characterization techniques. Successful synthesis was initially assessed by UV–Visible spectroscopy, while X-ray diffraction (XRD) was used to determine structural and crystalline properties. FTIR analysis identified the functional groups potentially responsible for nanoparticle reduction, capping, and stabilization. Particle size and morphology were studied using transmission electron microscopy (TEM) (JEOL, Tokyo, Japan) [25]. It should be emphasized that TEM and DLS measure fundamentally different particle attributes. TEM determines the primary particle size under dry-state conditions, whereas DLS measures the hydrodynamic diameter of particles dispersed in liquid, which is influenced by solvation layers and agglomeration. Therefore, the two techniques should be considered complementary rather than interchangeable.

#### 2.4.1. UV–Visible Spectroscopy

The biosynthesized ZnO and CuO nanoparticles were investigated for their optical properties using UV–Visible spectroscopy. The nanoparticles were synthesized using the conditions described above, and distilled water was used as a blank reference. The spectral scans were carried out using a UV–Vis spectrophotometer in the range of 200–700 nm, with data points recorded at 10 nm intervals. The absorbance peaks supported the formation of nanoparticles, exhibiting characteristic optical absorption behavior [26].

#### 2.4.2. X-Ray Diffraction (XRD) Analysis

X-ray diffraction (XRD) was employed to determine crystallinity, phase composition, and crystallite size. Measurements were conducted using a RIGAKU DMAX2500 diffractometer (Akishima-shi, Tokyo, Japan) with Cu Kα radiation (λ = 1.5406 Å) at 40 kV and 40 mA. patterns were acquired over a 2θ range of 20–80° at 5°/min. Peak positions were compared with standard JCPDS data to verify phase identity and purity. Mean crystallite size was calculated using the Debye–Scherrer equation [27].

#### 2.4.3. Fourier-Transform Infrared (FTIR) Spectroscopy

FTIR spectroscopy was used to identify different functional groups in the nanoparticles and to elucidate the involvement of functional groups for the reduction and stabilization of ZnO and CuO from *Moringa oleifera* extract. Nanoparticles were synthesized by mixing *Moringa oleifera* extract with metal precursors under controlled conditions. FTIR spectroscopy using the Bomem MB157S model was employed from 4000 to 400 cm^−1^ at room temperature. Absorption bands detected were attributed to phenols, flavonoids, and other biomolecules that help stabilize nanoparticles [28].

#### 2.4.4. Transmission Electron Microscopy (TEM)

The morphology and particle size were studied using a JEM-100CXII transmission electron microscope (JEOL, Tokyo, Japan) operated at 120 kV. Transmission electron microscopy was used to examine nanoparticle morphology and estimate particle-size distribution. Nanoparticle suspensions were deposited onto carbon-coated copper grids and dried at room temperature before imaging [29].

### 2.5. Cell Culture and Antiviral Assay

Authenticated viral reference materials and established cell lines were obtained through Nawah Scientific (Cairo, Egypt; ISO/IEC 17025-accredited laboratory), which provided the required biological materials and technical support according to standard virological procedures. The viral strains and cell lines used in this study represent established research materials with traceable sources and were not newly isolated, generated, or derived specifically for this study. Madin-Darby Canine Kidney (MDCK) and BHK-21 cells were cultured in Dulbecco’s Modified Eagle Medium (DMEM) with 10% fetal bovine serum (FBS) and 1% antibiotic–antimycotic solution. The cells were incubated at 37 °C in humidified 5% CO_2_ until a monolayer formed.

MDCK cells were used as the laboratory-validated screening platform routinely employed for comparative antiviral susceptibility assays under standardized experimental conditions (against CMV and rotavirus) rather than for propagating the viruses themselves based on the observation of cytopathic effect (CPE) in accordance with the laboratory’s approved protocol; similarly, BHK-21 cells were used for human papillomavirus (HPV) assays, rather than for propagating the virus itself, as previously indicated. The antiviral activity of the synthesized nanoparticles was initially evaluated using a crystal violet cytopathic effect (CPE) inhibition assay as a screening approach to assess their ability to protect infected cells from virus-induced cytopathic damage [30]. This method provides a rapid and reproducible preliminary evaluation of antiviral efficacy before more specific molecular assays. For some assays, viral inocula were prepared at a multiplicity of infection (MOI) of 0.01–0.1 depending on the assay condition. Cells were infected and allowed to adsorb for 1 h at 37 °C with gentle rocking, followed by removal of the inoculum and addition of maintenance medium containing the tested nanoparticles. Infected cultures were then incubated for a further 48–72 h and used to determine antiviral activity, as indicated by inhibition of virus-induced cytopathic effect (CPE) in comparison with untreated infected controls.

All cytotoxicity and antiviral assays were performed under standard laboratory lighting in a biosafety cabinet and CO_2_ incubator (non-UV, ambient indoor light); light exposure during handling was not systematically controlled or varied as an independent variable.

#### 2.5.1. Cytotoxicity Assay

Before antiviral testing, we screened the synthesized nanoparticles for potential cytotoxic effects on host cells. Nanoparticles (0.1–1000 µg/mL) were two-fold serially diluted in serum-free DMEM and added to cell monolayers in 96-well plates. Cells were seeded in 96-well plates at a density of 1 × 10^4^ cells/well and allowed to grow to approximately 80–90% confluency prior to treatment. Following vortexing, the serum-free DMEM used to disperse the nanoparticles was sonicated for 15–30 min at room temperature to ensure a homogeneous suspension. Nanoparticle suspensions were aseptically prepared and filtered through a 0.22 µm syringe filter before exposure to cells. All assays were performed in three independent biological experiments, each conducted with three technical replicates. After 72–96 h of incubation, cell viability was estimated by crystal violet staining. The CC_50_ value, or the concentration causing a 50% reduction in viability, was calculated using Prism (GraphPad Software, version 5.0d; GraphPad Software, Inc., San Diego, CA, USA) [31]. Negative controls (untreated cells) and positive controls (cells treated with a known cytotoxic agent) were included in all assays to determine the relative toxicity of nanoparticles.

#### 2.5.2. In Vitro Antiviral Activity Assessment

The cells were washed with phosphate-buffered saline (PBS) and subsequently infected with viral inocula at a multiplicity of infection (MOI) of 0.01–0.1, the MOI that would induce a clear cytopathic effect (CPE) in uncomplicated control wells during the incubation period. Adsorption of the viruses was performed for 1 h at 37 °C with gentle agitation. After incubation, the inoculum was discarded, the cells were washed with PBS, and fresh maintenance medium containing the tested nanoparticles was added immediately after infection. The incubations of infected cultures were performed under standard conditions (37 °C, 5% CO_2_) for virus-specific times: 72 h for HCMV, 48 h for rotavirus, and 48–72 h for HPV. Antiviral activity was then estimated by the reduction in virus-induced CPE compared to the untreated infected control. After virus adsorption, infected cells were treated with various nanoparticle concentrations. Plates were incubated at 37 °C with 5% CO_2_ and checked daily for CPE using an inverted microscope. At the end of incubation, cells were fixed and stained with 0.03% crystal violet. Plates were washed with distilled water to remove excess stain and allowed to air-dry [30]. Negative controls (untreated cells) and positive controls (cells treated with an antiviral agent) were included for comparison.

Antiviral activity was estimated by the reduction in virus-induced CPE compared to the untreated infected control, using crystal violet-based cell viability as a surrogate endpoint. This CPE/viability-based readout is an indirect measure of antiviral efficacy: it reflects preservation of the host-cell monolayer rather than direct quantification of viral genome replication, transcription, or progeny virion production.

#### 2.5.3. Assessment of Antiviral Activity

Optical density (OD) at 570 nm was measured using LisaScan EM microplate reader (Erba Lachema s.r.o., Brno, Czech Republic). Nonlinear regression analysis was used to calculate the IC_50_ value, defined as the nanoparticle concentration producing 50% inhibition of virus-induced cytopathic effect (CPE). The percentage of antiviral inhibition was calculated as follows: Inhibition (%) = [(OD treated infected cells − OD infected control cells)/(OD uninfected control cells − OD infected control cells)] × 100. The selectivity index (SI), defined as the ratio of each nanoparticle’s CC_50_ to IC_50_, was used to evaluate antiviral activity and safety. As a result, greater SI values were considered indicative of a larger in vitro selectivity window.

### 2.6. Computational Prediction of Protein–Ligand Interactions Using Boltz-2

To perform the docking process, SMILES codes for the flavonoid ligands were used as input in addition to the FASTA sequences for each target (PDB: 1YYP, 1SLQ, and 4GIZ) as a requirement for Boltz-2 [21] to predict and score the binding of ligand–protein complexes. The PDB files obtained from the PDB website [32,33] were used as templates to enhance the model and predict correct co-folding with more precise IC_50_ and likelihood [34]. To summarize, the model performed 3 recycling steps and 20 sampling steps using a step scale of 1.64 [34]. Computational IC_50_ values are generated as incidental outputs of the Boltz-2 model but were not used to infer antiviral potency. Instead, candidate ranking was based strictly on the affinity likelihood scores, which we consider the more accurate metric for binding probability. Both the computational IC_50_ and affinity likelihood outputs are reported solely for relative ranking purposes and should not be interpreted as quantitative measures of experimental antiviral activity.

### 2.7. Molecular Dynamics Simulation Protocol

The simulations were performed with GROMACS 2024.2 using the OPLS-AA force field [34] for standard residues and LigParGen [35] to generate the ligand parameters. The solvation model used was TIP3P with a 1.0 nm distance from protein to the box edge. Na^+^/Cl^−^ ions were used to neutralize the system at 0.15 M concentration. Energy minimization was performed using the steepest descent algorithm [36,37] until the maximum force fell below 1000 kJ/mol/nm. The system was equilibrated by two phases: the NVT phase stabilized the temperature at 300 K using a V-rescale thermostat, followed by NPT to equilibrate the system pressure at 1.0 bar via the Parrinello-Rahman barostat. Both NVT and NPT were held under positional restraints on heavy atoms for 100 ps before running a 150 ns NPT MD simulation with a 2fs time step. All simulation phases used PBC. To study the dynamical stability of the ligand’s binding pose predicted by Boltz-2, an MD simulation study was performed to analyze the root mean square deviation of the ligand from the starting position using RMSD and by also measuring the short-range interaction energies and Lennard-Jones (LJ) energies. To study the protein stability, we measured the root mean square fluctuation of the amino acids using RMSF, radius of gyration (RoG), and Solvent Accessible Surface Area (SASA) [38].

### 2.8. Statistical Analysis

All assays were performed in three independent biological experiments, each conducted with three technical replicates. CC_50_ and IC_50_ values were estimated by nonlinear regression analysis of dose–response curves generated using Prism (GraphPad Software, version 5.0d; GraphPad Software, Inc., San Diego, CA, USA). The reported values represent the estimates obtained from three independent experiments. The selectivity index (SI) was calculated as CC_50_/IC_50_.

## 3. Results

### 3.1. Zinc Oxide Nanoparticle Characterization

**Figure 1:**
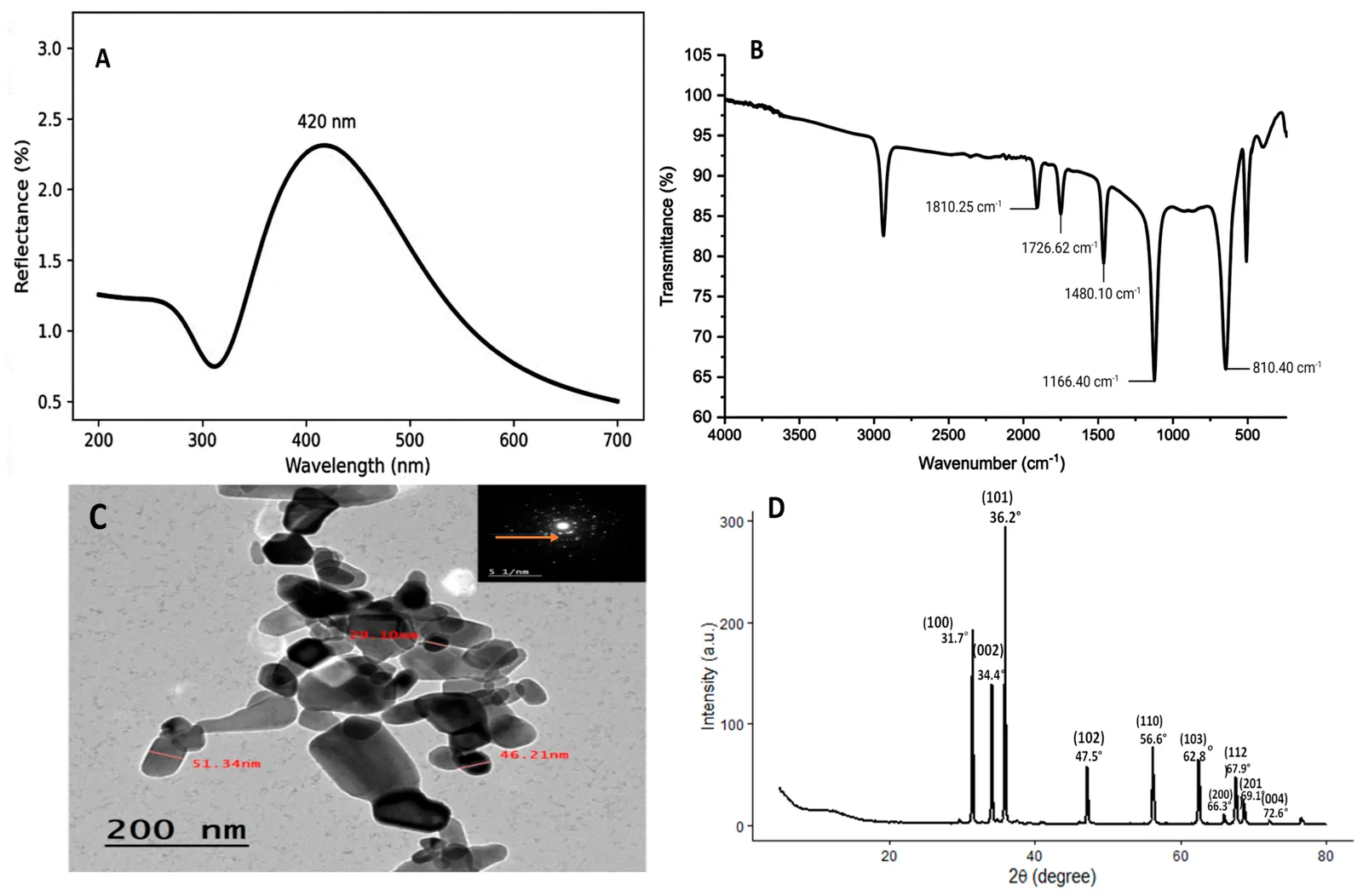
Physicochemical characterization of green-synthesized ZnO nanoparticles (ZnO-NPs) prepared using *Moringa oleifera* leaf extract. (**A**) UV–Vis absorption spectrum of green-synthesized zinc oxide nanoparticles (ZnO-NPs) prepared using *Moringa oleifera* leaf extract shows a characteristic absorption peak at approximately 420 nm, confirming the formation of ZnO-NPs. (**B**) FTIR spectrum of green-synthesized zinc oxide nanoparticles (ZnO-NPs) prepared using *Moringa oleifera* leaf extract. FTIR spectra were recorded in the range 4000–400 cm^−1^, confirming the presence of phytochemical functional groups associated with nanoparticle synthesis and stabilization. (**C**) Transmission electron microscopy (TEM) image of green-synthesized zinc oxide nanoparticles (ZnO-NPs). The nanoparticles exhibit predominantly quasi-spherical to slightly elongated morphologies with particle sizes ranging from approximately 29 to 51 nm and moderate agglomeration. The inset shows the selected area electron diffraction (SAED) pattern, displaying concentric diffraction rings characteristic of the polycrystalline hexagonal wurtzite ZnO structure. The orange arrow indicates a representative diffraction ring in the SAED pattern. (**D**) X-ray diffraction (XRD) pattern of green-synthesized zinc oxide nanoparticles (ZnO-NPs), showing the characteristic diffraction peaks of the crystalline hexagonal wurtzite structure (ICDD PDF No. 36-1451). The diffraction peaks indicate a crystalline ZnO phase, confirming the successful formation of ZnO-NPs by green synthesis using *Moringa oleifera* extract.

The spectra display a significant absorption peak at approximately 420 nm, attributed to the band gap transition of the hexagonal wurtzite phase of ZnO, consistent with previous reports. This peak, redshifted by about 28 nm compared to bulk ZnO, indicates a reduced optical band gap. This change is associated with the creation of a donor level in the band gap, The redshift of the absorption peak signals a changed electronic structure in the nanoparticles, suggesting that both size and lattice defects influence their optical properties, as shown in Figure 1A [39].

The FT-IR spectrum in Figure 1B provides further details regarding the surface chemistry of nanomaterials. In comparison to Zn nanoparticles, fewer absorption bands are observed. Moving to specific features, the strong band at 1810.25 cm^−1^ is assigned to C=O stretching vibrations typical of carbonyl groups in carboxylic acids or their anhydrides. Additionally, another prominent band appears at 1726.62 cm^−1^, representing C=O stretching in aldehydes, ketones, or esters. Medium absorptions at 1480.10 cm^−1^ and 1166.40 cm^−1^ further indicate C–H bending of alkanes and C–O stretching of ethers or esters, respectively. A peak at 810.40 cm^−1^ corresponds to C–H bond bending in aromatic systems. These features indicate surface functional groups that may enhance the chemical reactivity and stabilization of ZnO nanoparticles in aqueous media.

The TEM image of the green-synthesized ZnO-NPs indicated that the ZnO-NPs were mainly quasi-spherical to slightly elongated nanoparticles with particle sizes around 29–51 nm. The nanoparticles show slight agglomeration, which is a common feature of biosynthesized ZnO nanoparticles due to their high surface energy and considerable interparticle forces. The inset shows a selected area electron diffraction (SAED) pattern showing the concentric diffraction rings of this polycrystalline structure. These findings align with earlier investigations, where similar nanoparticle sizes and larger z-average diameters were reported. This agreement confirms that the synthesis approach successfully controlled the growth and particle shape as shown in Figure 1C [39].

The X-ray diffraction (XRD) pattern of ZnO nanoparticles in Figure 1D verifies the formation of a wurtzite structure. The observed diffraction peaks at approximately 31.9°, 34.6°, 36.4°, 47.3°, 56.8°, 62.3°, 66.5°, 67.7°, 69.3°, and 72.4° were indexed to the (100), (002), (101), (102), (110), (103), (200), (112), (201), and (004) planes, respectively, consistent with the hexagonal wurtzite ZnO structure (ICDD PDF No. 36-1451).

### 3.2. Copper Oxide Nanoparticle Characterization

**Figure 2:**
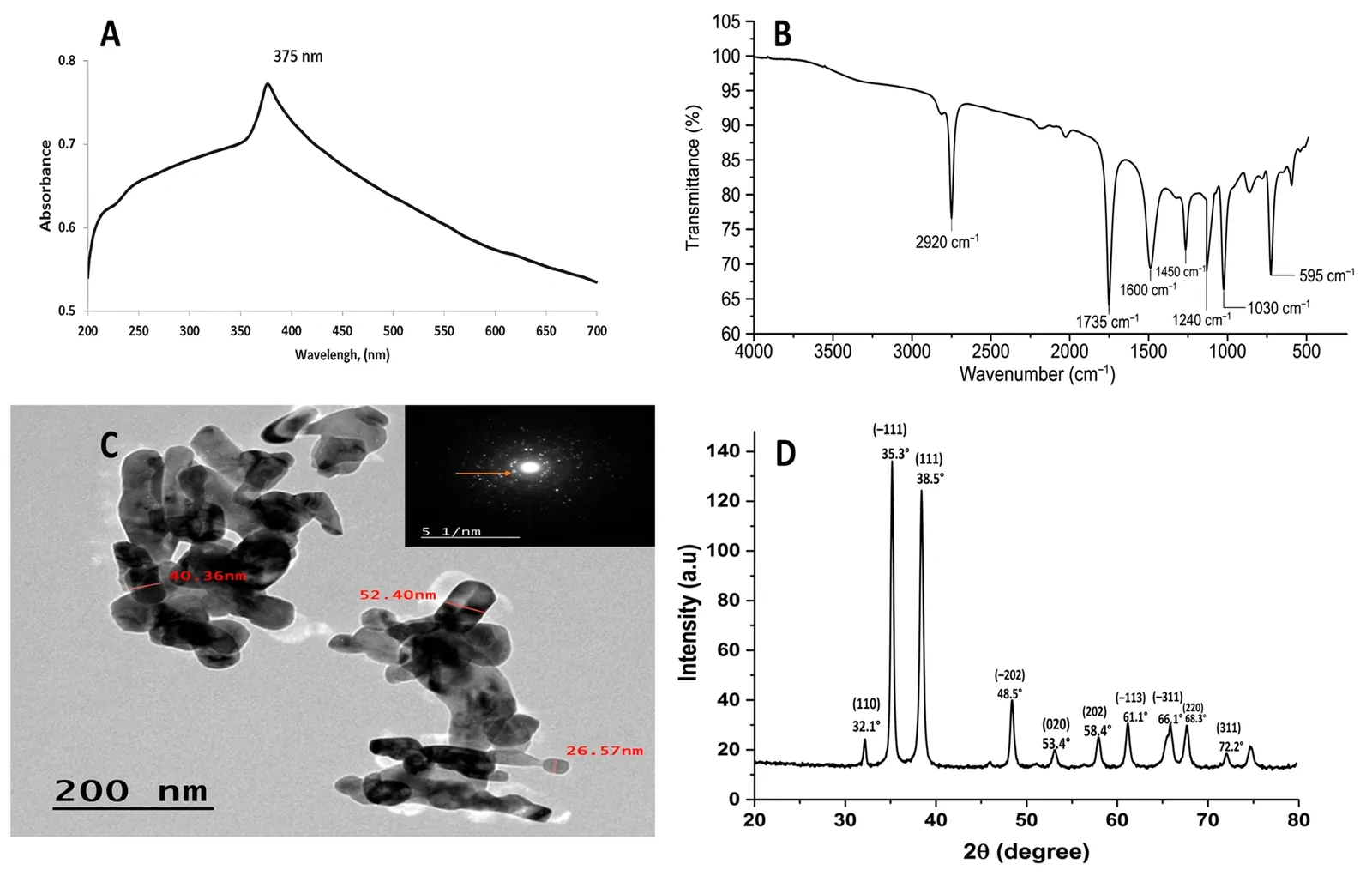
Physicochemical characterization of green-synthesized CuO nanoparticles (CuO-NPs) prepared using *Moringa oleifera* leaf extract. (**A**) UV–Vis absorption spectrum of green-synthesized copper oxide nanoparticles (CuO-NPs) shows a characteristic surface plasmon resonance band peaking at approximately 375 nm, confirming CuO-NP formation. (**B**) Fourier-transform infrared (FTIR) spectrum of green-synthesized copper oxide nanoparticles (CuO-NPs), showing the characteristic vibrational bands of phytochemical functional groups involved in nanoparticle formation and stabilization, together with the Cu–O stretching vibration confirming CuO nanoparticle formation. (**C**) Transmission electron microscopy (TEM) image of green-synthesized copper oxide nanoparticles (CuO-NPs). The nanoparticles exhibit quasi-spherical to irregular morphologies with particle sizes ranging from approximately 26 to 52 nm. The inset shows the selected area electron diffraction (SAED) pattern, confirming the polycrystalline crystalline structure of the synthesized monoclinic CuO nanoparticles. The orange arrow indicates a representative electron diffraction spot in the SAED pattern. (**D**) X-ray diffraction (XRD) pattern of green-synthesized CuO nanoparticles showing the characteristic diffraction peaks of the monoclinic CuO (tenorite) crystal structure, consistent with the ICDD/JCPDS PDF No. 48-1548 reference pattern.

The green-synthesized CuO nanoparticles were characterized for their optical properties by UV at 200 to 700 nm at 37 °C. As shown in Figure 2A, UV–Visible spectra of (CuO-NPs) exhibited a peak at 375 nm, confirming nanoparticle formation by the green approach. Copper SPR was observed at 375 nm, indicating excitation of surface plasmon vibrations in colloidal copper oxide nanoparticles in the 300–390 nm region [40]. These optical features are critical indicators in nanoparticle characterization and corroborate structural data.

The FTIR analysis was used to identify the bio-parts involved in synthesizing (CuO-NPs) during the reaction. The FTIR spectrogram, shown in Figure 2B, of the green-synthesized CuO nanoparticles exhibited a broad absorption band around 3400 cm^−1^, corresponding to O–H stretching vibrations of phenolic compounds and adsorbed water molecules derived from the *Moringa oleifera* extract. Weak bands near 2920 cm^−1^ were assigned to aliphatic C–H stretching vibrations. The absorption band around 1735 cm^−1^ was attributed to C=O stretching of carbonyl-containing phytochemicals, while bands between 1600 and 1450 cm^−1^ were associated with aromatic C=C vibrations. The absorption band in the 1030–1240 cm^−1^ region corresponded to C–O stretching vibrations of alcohols and phenolic compounds. Most importantly, the characteristic absorption band observed at approximately 595 cm^−1^ was assigned to Cu–O stretching vibrations, confirming the formation of copper oxide nanoparticles.

Transmission electron microscopy (TEM) image of green-synthesized CuO nanoparticles showing predominantly quasi-spherical to irregularly shaped nanoparticles with particle sizes ranging from approximately 26 to 52 nm. The nanoparticles exhibit moderate agglomeration, which is commonly observed in biosynthesized CuO nanoparticles due to their high surface energy and strong interparticle interactions. The inset presents the selected area electron diffraction (SAED) pattern, displaying well-defined concentric diffraction rings characteristic of a polycrystalline structure [41].

The crystalline structure and orientation of CuO nanoparticles were characterized by XRD analysis, as shown in Figure 2D. The XRD pattern exhibited characteristic reflections at approximately 2θ = 32.1°, 35.3°, 38.5°, 48.5°, 53.4°, 58.4°, 61.1°, 66.1°, 68.3°, and 72.2°, which were indexed to the (110), (−111), (111), (−202), (020), (202), (−113), (−311), (220), and (311) crystallographic planes of monoclinic CuO (ICDD/JCPDS PDF No. 48-1548). No characteristic diffraction peak corresponding to metallic copper at 43.3° was observed, indicating that the synthesized material predominantly consists of crystalline CuO nanoparticles rather than metallic Cu. Phase purity and crystal morphology are critical for optimising photocatalytic, antimicrobial and electronic applications. These reflections confirm the formation of crystalline CuO nanoparticles, indicating successful green synthesis [42].

### 3.3. Antiviral Activity

#### 3.3.1. Antiviral Activity Against Human Papilloma Virus

Table 1 summarizes CC_50_, which are the concentrations of ZnO and CuO nanoparticles that induce cytotoxic effects in 50% of cells, and IC_50_, representing the concentrations needed to achieve 50% inhibition of viral replication. The selectivity index (SI) against human papillomavirus is shown in a separate column, calculated from the CC50 and IC50 values.

**Figure 3:**
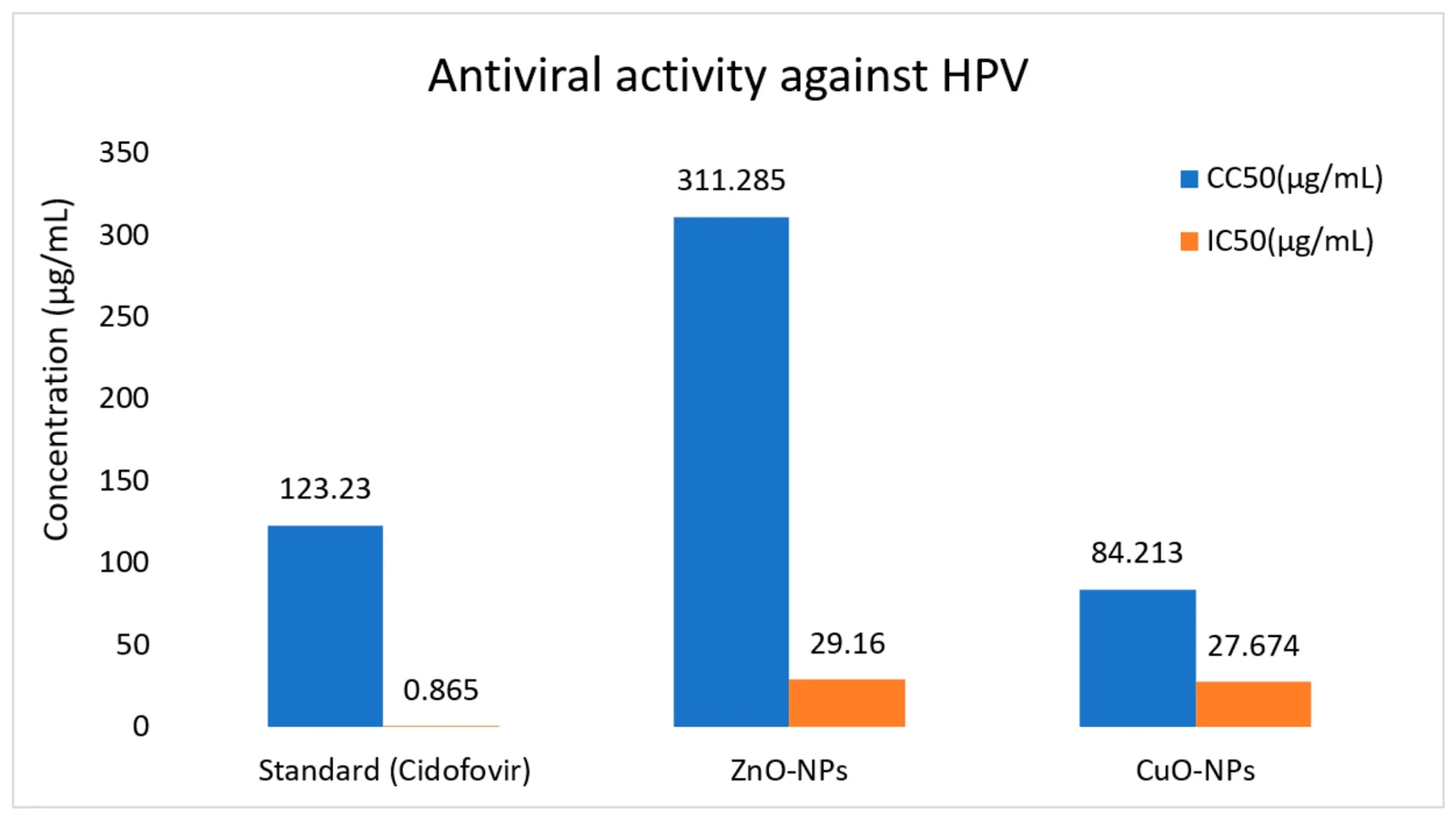
Cytotoxicity (CC_50_) and antiviral activity (IC_50_) of green-synthesized ZnO-NPs and CuO-NPs across HPV. CC_50_ and IC_50_ values were obtained by nonlinear regression analysis of dose–response curves generated from three independent biological experiments using GraphPad Prism. SI values are presented in Table 1.

**Figure 4:**
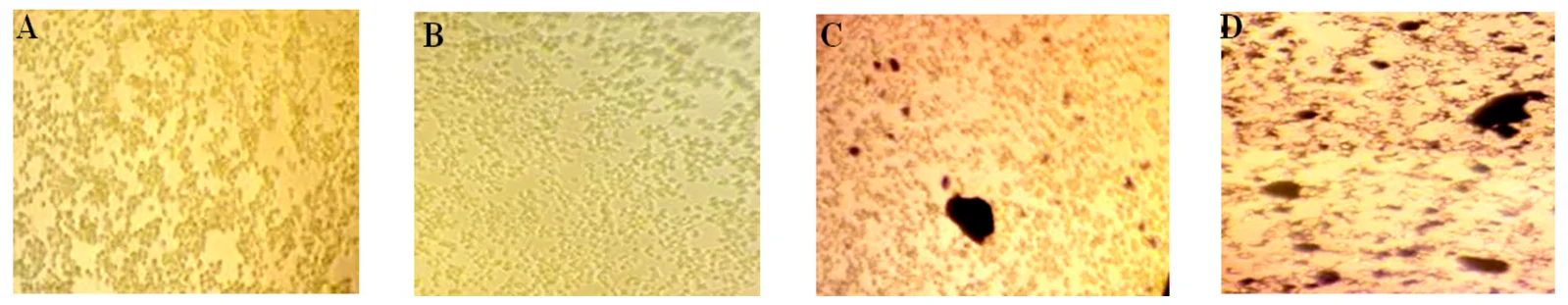
Antiviral activity and cytotoxicity of green-synthesized ZnO nanoparticles and CuO nanoparticles were tested against human papillomavirus (HPV), HPV16 in BHK-21 cells. (**A**) ZnO-NPs produced a cytopathic effect on HPV-infected cells. (**B**) ZnO-NPs caused cytotoxic effects on non-infected cells. (**C**) CuO-NPs produced a cytopathic effect on HPV-infected cells. (**D**) CuO-NPs caused cytotoxic effects on non-infected cells. Cell viability and cytopathic effects were measured with the crystal violet assay after (48–72) h of incubation.

ZnO nanoparticles demonstrated an acceptable level of antiviral activity, with a high CC_50_ (311.285 µg/mL) and a moderate IC_50_ (29.160 µg/mL). This resulted in an SI of 10.67, which indicates acceptable cellular safety. CuO nanoparticles, in contrast, showed slightly lower IC_50_ (IC_50_ = 27.674 µg/mL) but were markedly more cytotoxic (CC_50_ = 84.213 µg/mL). This led to a low SI of 3.04. The antiviral screening demonstrated distinct biological profiles for the tested nanoparticles. Although both ZnO-NPs and CuO-NPs exhibited anti-HPV activity, ZnO-NPs showed a markedly more favorable selectivity profile, reflecting an improved balance between antiviral efficacy and host-cell compatibility. In contrast, the lower selectivity index of CuO-NPs indicates that their antiviral effect was accompanied by greater cytotoxicity, thereby reducing their therapeutic window. Compared with the reference antiviral drug, ZnO-NPs demonstrated promising antiviral potential while maintaining substantially lower toxicity toward BHK-21 cells under the present experimental conditions as seen in Figure 3 and Figure 4.

#### 3.3.2. Antiviral Activity Against Rotavirus

Table 2 summarizes the CC_50_ values, defined as the concentrations of ZnO and CuO nanoparticles that induce cytotoxic effects in 50% of the cells, and the IC_50_ values, which correspond to the concentrations required to achieve 50% inhibition of viral replication. The selectivity index (SI) against rotavirus is shown in a separate column, calculated from the CC_50_ and IC_50_ values.

**Figure 5:**
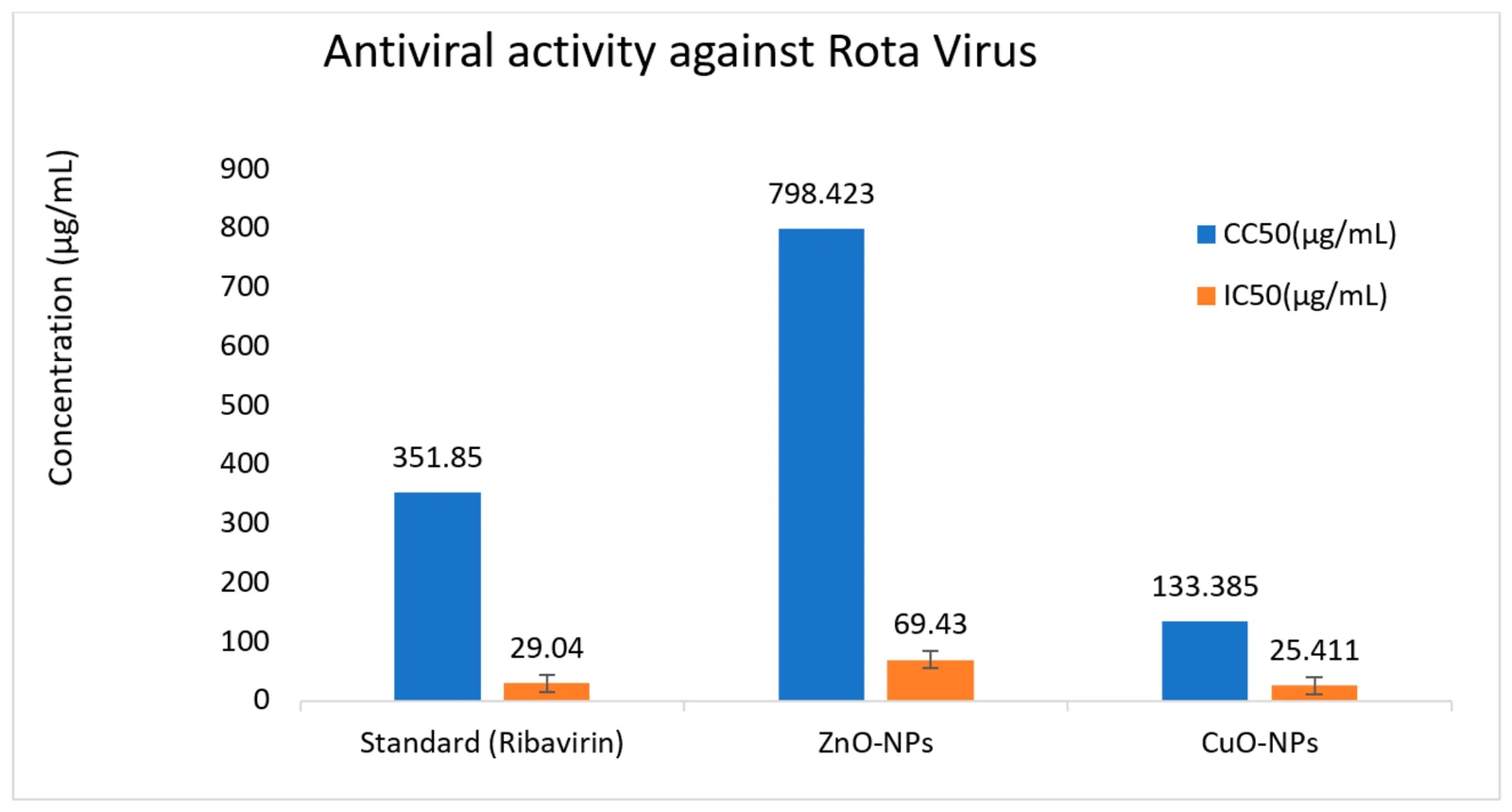
Cytotoxicity (CC_50_) and antiviral activity (IC_50_) of green-synthesized ZnO-NPs and CuO-NPs across rotavirus. CC_50_ and IC_50_ values were obtained by nonlinear regression analysis of dose–response curves generated from three independent biological experiments using GraphPad Prism. SI values are presented in Table 2.

**Figure 6:**
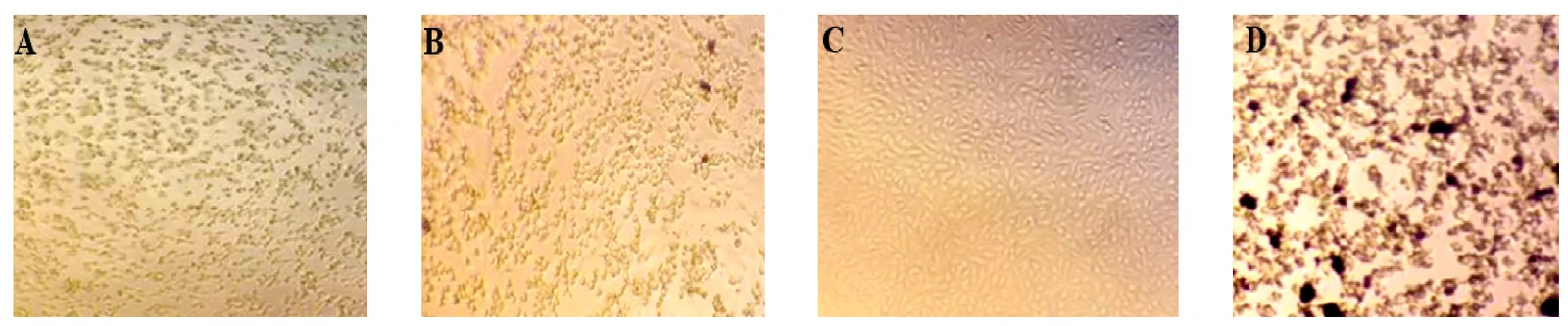
The antiviral activity and cytotoxicity of green-synthesized ZnO nanoparticles (ZnO-NPs) and CuO nanoparticles (CuO-NPs) against rotavirus. (**A**) The cytopathic effect of ZnO-NPs on rotavirus-infected cells. (**B**) The cytotoxic effect of ZnO-NPs on non-infected cells. Transitioning to the analysis of copper oxide nanoparticles, (**C**) shows the cytopathic effect of (CuO-NPs) on rotavirus-infected cells, and (**D**) the cytotoxic effect of CuO-NPs on non-infected cells. In all cases, cell viability and cytopathic effects were estimated with MDCK cells using the crystal violet assay after 48 h of incubation.

To elaborate, the CC_50_ value for ZnO nanoparticles was high (798.42 µg/mL), suggesting low cytotoxicity, while the IC_50_ was 69.43 µg/mL, resulting in an SI of 11.49, which represents good selectivity for antivirals [35]. In comparison, CuO nanoparticles were more effective against the virus (IC_50_ = 25.411 µg/mL) but also more cytotoxic (CC_50_ = 133.385 µg/mL), resulting in an SI of 5.24. ZnO-NPs demonstrated the most favorable antiviral profile against rotavirus, exhibiting a superior balance between antiviral efficacy and cytocompatibility compared with CuO-NPs. Although both nanomaterials reduced virus-induced cytopathic effects, the markedly higher selectivity index of ZnO-NPs indicates a wider therapeutic window and suggests that their antiviral activity was achieved with lower host-cell toxicity. In contrast, the lower selectivity index of CuO-NPs reflects greater cytotoxicity relative to their antiviral effect, potentially limiting their biological applicability under the present experimental conditions, as seen in Figure 5 and Figure 6.

#### 3.3.3. Antiviral Activity Against Cytomegalovirus (CMV)

Table 3 presents CC_50_ values, which indicate the concentrations of ZnO and CuO nanoparticles that cause cytotoxicity in 50% of cells. It also lists IC50 values, which represent the concentrations needed to inhibit viral replication by 50%. The selectivity index (SI) against cytomegalovirus (CMV) is shown in a separate column, calculated from the CC_50_ and IC_50_ values.

**Figure 7:**
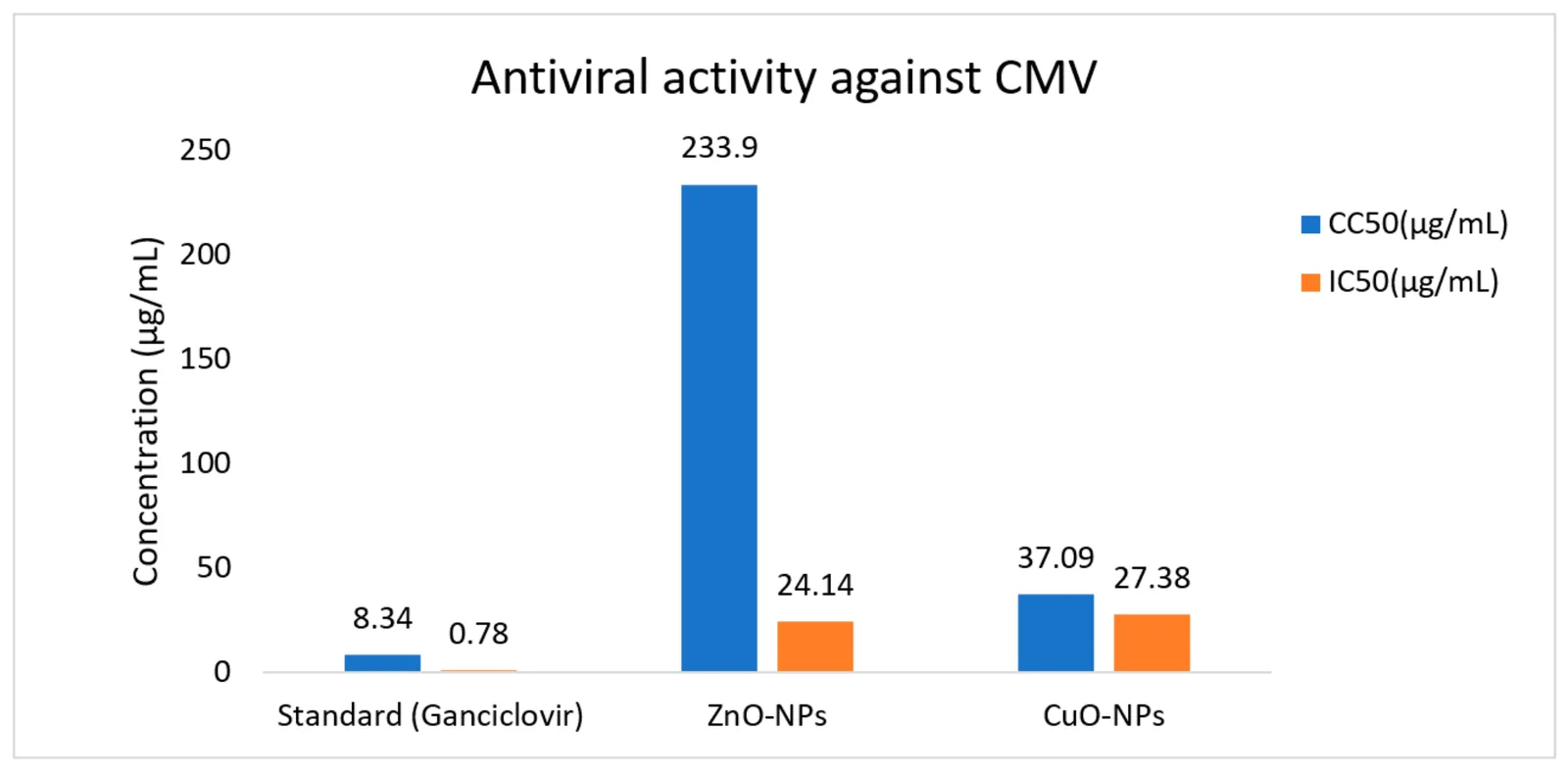
Cytotoxicity (CC_50_) and antiviral activity (IC_50_) of green-synthesized ZnO-NPs and CuO-NPs across CMV. CC_50_ and IC_50_ values were obtained by nonlinear regression analysis of dose–response curves generated from three independent biological experiments using GraphPad Prism. SI values are presented in Table 3.

**Figure 8:**
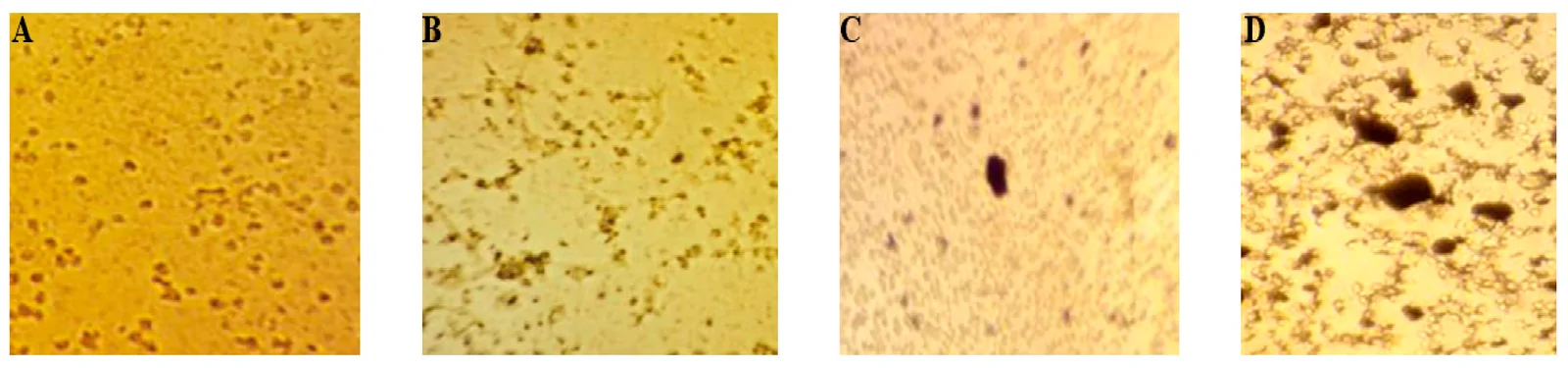
Antiviral activity and cytotoxicity of green-synthesized ZnO nanoparticles (ZnO-NPs) and CuO nanoparticles (CuO-NPs) against cytomegalovirus (CMV). (**A**) Cytopathic effect of ZnO-NPs on CMV-infected cells. (**B**) Cytotoxic effect of ZnO-NPs on non-infected MDCK cells. (**C**) Cytopathic effect of CuO-NPs on CMV-infected cells. (**D**) Cytotoxic effect of CuO-NPs on non-infected MDCK cells. Cell viability and cytopathic effects were estimated using the crystal violet assay after 72 h of incubation.

The data revealed divergent antiviral activities of ZnO and CuO nanoparticles against human cytomegalovirus (CMV), as shown in Figure 7 and Figure 8. The CC_50_ for ZnO nanoparticles was 233.91 µg/mL. The IC50 was 24.14 ug/mL, giving a selectivity index of 10.57. This indicates high antiviral potency with acceptable cytotoxicity. In contrast, CuO nanoparticles had a lower SI. CuO-NPs showed a CC50 of 37.09 ug/mL and an IC50 of 27.38 ug/mL, i.e., comparable antiviral potency to ZnO-NPs but at a far lower cytotoxic threshold. The SI for copper (1.35) indicates non-selective action with greater cytotoxicity. Accordingly, ZnO-NPs showed the more favourable anti-CMV profile, combining CPE inhibition with lower host-cell toxicity. The higher selectivity index of ZnO-NPs relative to CuO-NPs indicates a wider in vitro selectivity window, i.e., the antiviral effect was obtained at concentrations further below the cytotoxic threshold. By contrast, the low selectivity index for CuO nanoparticles suggests that cytotoxicity may considerably limit their antiviral activities under these experimental conditions. In summary, these results reveal that ZnO-NPs are the superior candidate for downstream evaluation against CMV infection.

#### 3.3.4. Molecular Docking via Boltz-2

The antiviral potential of selected phytochemicals was evaluated through computational prediction of IC_50_ values and binding likelihood against key viral protein targets, as presented in Table 4. Comparative analysis of ligand–protein interactions provide insight into the relative inhibitory efficiency and binding affinity of the investigated compounds.

**Figure 9:**
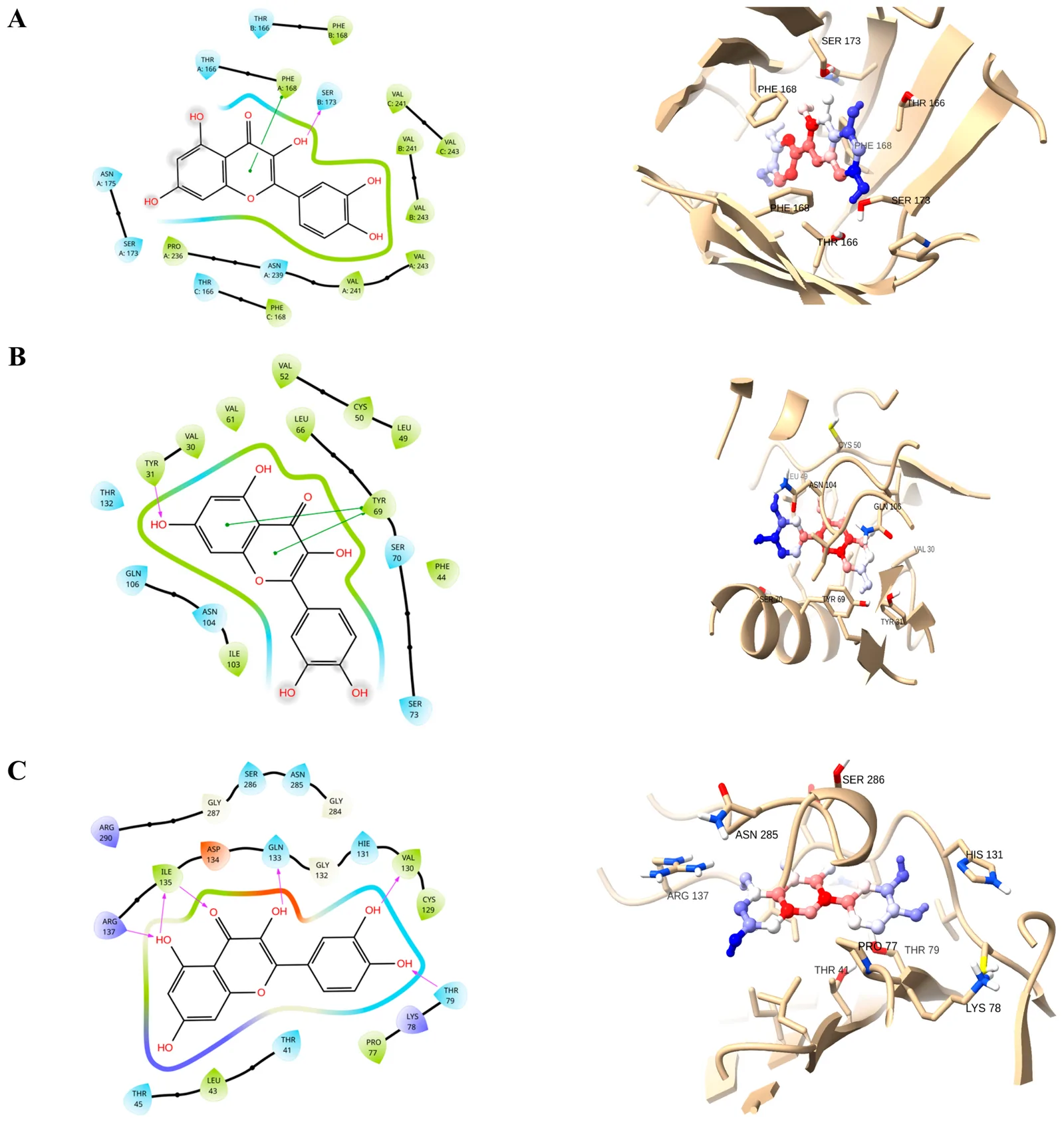
3D conformational analysis and 2D interaction map of quercetin. Predicted 3D binding orientations of quercetin and 2D interaction map within the active site grooves of (**A**) rotavirus VP5* (PDB 1SLQ), (**B**) HPV E6 Oncoprotein (PDB 4GIZ), and (**C**) CMV UL44 processivity factor (PDB 1YYP). 3D ligand is colored by B-factor. In 2D maps, green represents π-interactions and purple represents hydrogen bonds.

Co-folding of the ligand SMILES strings with the target sequences produced the predicted pIC_50_ and binding likelihood values presented in Table 4. The binding mode of quercetin with the trimeric structure of rotavirus VP5* as seen in Figure 9A is characterized by a hydrogen bond formed between the 3-hydroxyl group of the quercetin scaffold and the side chain of Ser B:173, as well as the stabilization of the aromatic ring by π-interaction with the phenyl ring of Phe A:168. The 3′,4′-dihydroxy-substituted B-ring causes hydrophobic interactions with Val A:241, Val A:243, Val B:241, Val B:243, and Val C:243 creating a continuous van der Waals surface. Figure 9B highlights the interaction profile between quercetin and the human papillomavirus (HPV) oncoprotein E6 binding cleft. The 2D interaction map shows that a hydrogen bond is formed between the side-chain hydroxyl group of Tyr 31 and the 7-hydroxyl group of the quercetin A-ring. The chromone core of quercetin participates in T-shaped interactions with the aromatic phenol ring of Tyr 69. The ligand is encapsulated by a hydrophobic envelope, composed of Val 30, Leu 49, Cys 50, Val 52, Val 61, Leu 66, and Ile 103. Figure 9C shows the interaction between quercetin and the human cytomegalovirus (CMV) DNA polymerase processivity factor, UL44. The 2D interaction map shows that the 5-hydroxyl group and the 4-carbonyl oxygen both form hydrogen bonds with Ile 135. Simultaneously, the 5-hydroxyl group participates in a hydrogen bond with Arg 137. The 3-hydroxyl group of the C-ring forms a hydrogen bond with the side chain of Gln 133; the 3′-hydroxyl group interacts with Val 130; and the 4′-hydroxyl group forms a hydrogen bond with Thr 79. The ligand is supported by a hydrophobic environment, Pro 77, Val 130, Cys 129, His 131, Asp 134, and Leu 43.

#### 3.3.5. Molecular Dynamics (MD) Simulation Analysis

**Figure 10:**
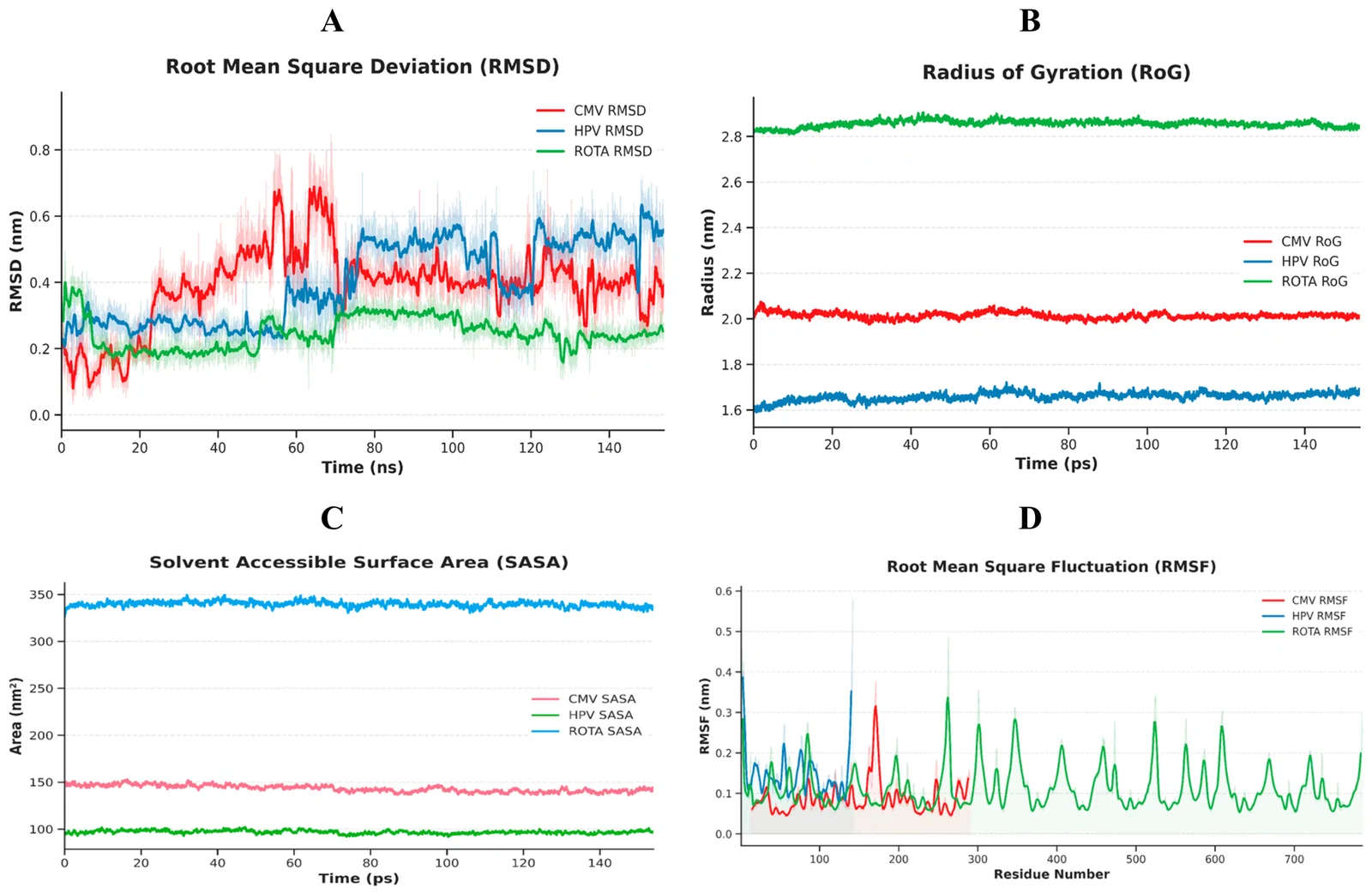
Structural stability and conformational dynamics of viral target proteins during 150 ns Molecular Dynamics (MD) simulations. The analysis focuses on CMV UL44 processivity factor (PDB 1YYP), HPV E6 Oncoprotein (PDB 4GIZ), and rotavirus VP5* (PDB 1SLQ). (**A**) Root mean square deviation (RMSD) of the ligand atoms relative to the alpha-carbon backbone. (**B**) Radius of gyration (RoG) depicts the structural compactness over time. (**C**) Solvent Accessible Surface Area (SASA) illustrates the exposure of the protein surface to the surrounding solvent. (**D**) Root mean square fluctuation (RMSF).

In Figure 10A which shows the RMSD of the ligand atoms (after least-squares fitting to the protein alpha-carbon backbone) for the different systems. CMV UL44 showed an initial increase, followed by stabilization at 0.41 nm after 70 ns. The same initial increase and stabilization time were observed for HPV E6 Oncoprotein, which stabilized at 0.58 nm after 70 ns, with a larger average deviation from the initially docked pose. For rotavirus VP5*, the RMSD value did not drift from the initial pose and remained around 0.2 nm throughout the simulation. Figure 10B,C show RoG and SASA for the three systems, with no obvious deviation from the initial frame throughout the simulation. Figure 10D shows the RMSF values for the residues of each system. CMV UL44 shows a spike of 0.31 nm at a loop region. HPV E6 shows a maximum RMSF of 0.32 nm at its N- and C-terminal residues. The trimeric structure of rotavirus VP5* shows high RMSF (~0.32 nm) at its N- and C-terminal residues. In addition to RMSD, RoG, SASA, and RMSF, the trajectories were further analyzed for short-range Lennard-Jones and Coulombic (electrostatic) interaction energies and hydrogen-bond occupancy over time; these additional metrics are presented in Supplementary, Figure S1 and Figure S2 respectively, and summarized in Section 4.

## 4. Discussion

Greenly-synthesized ZnO and CuO nanoparticles have emerged as promising antiviral agents due to their biocompatibility, eco-friendly production, and broad-spectrum activity against clinically important viruses. In this study, the prepared nanomaterials showed promising antiviral activities against HPV, rotavirus, and CMV, probably through mechanisms based on blocking viral structure, attachment, and replication to host cells.

The characterization results of ZnO and CuO nanoparticles confirmed successful green synthesis routes. These methods produce nanostructures with desirable particle sizes, morphologies, and phase purities. The observed UV-Vis spectral shifts and SPR peaks are consistent with previously reported bandgap and plasmonic properties, reflecting the influence of nanoparticle size, lattice defects, and surface chemistry. Additionally, FTIR and XRD analyses together confirm the presence of biomolecular capping and crystalline phase integrity. These features are essential for stability and reactivity. Similar observations have been reported for green-synthesized ZnO and CuO nanoparticles, whose physicochemical characteristics enable diverse applications, particularly in photocatalysis, antimicrobial therapy, and the biomedical field, as noted by other researchers [43].

These findings are also consistent with previous reports showing that nanoscale size, defect states, and biomolecular stabilization influence the optical and biological behavior of ZnO nanoparticles [44,45]. ZnO nanoparticles exhibited a more favorable antiviral selectivity profile than CuO nanoparticles against all tested viruses. Against HPV, ZnO nanoparticles showed lower cytotoxicity and a higher selectivity index than CuO nanoparticles indicating a broader therapeutic window under the tested in vitro conditions. Similar findings have been reported previously, including that ZnO nanoparticles may interfere with HPV-related viral attachment or capsid-associated processes. They accomplish this by inhibiting cell receptor binding and disruption of the capsid protein while maintaining biocompatibility [46]. In contrast, CuO nanoparticles exert antiviral effects mainly through oxidative damage and protein denaturation, mechanisms that may also increase host-cell toxicity [46]. Nevertheless, the antiviral endpoint in the present work was mainly based on cytopathic effect inhibition and cell viability; HPV-specific confirmation using viral DNA quantification, reporter expression, or viral protein detection is recommended.

A comparable pattern was observed against rotavirus. ZnO nanoparticles demonstrated a higher CC_50_ value and a selectivity index close to that of ribavirin, indicating selective antiviral activity with lower cytotoxicity, whereas CuO nanoparticles showed antiviral potency effects but at a higher risk of host-cell cytotoxicity due to their narrower therapeutic window [47]. Mechanistically, Earlier studies suggested that ZnO nanoparticles are reported to inhibit rotavirus by interacting with viral surface proteins, thereby hindering attachment and replication cycles, whereas copper oxide nanoparticles primarily induce antiviral activity through oxidative stress-mediated damage to viral proteins and nucleic acids; however, this is also associated with increased toxicity to host cells [48,49]. Together, these findings agree with previous studies on metal-based antiviral nanoparticles against enteric viruses, reinforcing ZnO’s promise as a safer antiviral agent. Consequently, ZnO nanoparticles appear more promising for antiviral treatments of rotavirus, demonstrating less cytotoxicity and more selective antiviral effects compared to copper oxide nanoparticles [50,51]. A similar contrast was observed against CMV, where ZnO-NPs again combined CPE inhibition with markedly lower cytotoxicity than CuO-NPs. Direct CMV-specific comparisons of these two materials are scarce, so this interpretation is extrapolated from studies in other viral systems. Interestingly, ZnO-NPs displayed superior selectivity with a significantly lower cytotoxicity than CuO-NPs supporting their greater antiviral potential under the investigated conditions. These results demonstrate greater potential for ZnO nanoparticles than copper oxide nanoparticles to inhibit CMV in vitro [51]. These discrepancies align with earlier studies which attributed the antiviral activity of ZnO nanoparticles to interference with viral envelope proteins and viral entry pathways. Importantly, this occurs without significant harm to host cells, whereas copper oxide nanoparticles also show broad-spectrum antiviral activity by causing oxidative damage and protein denaturation. However, their clinical use is limited by cytotoxicity [51]. The current findings reinforce the previous literature, positioning ZnO nanoparticles as a safer antiviral for CMV [52].

Overall, the differential selectivity and antiviral activity observed between ZnO and CuO nanoparticles likely reflects their distinct antiviral mechanisms and their specific interactions with viral and host-cell components [13,53]. ZnO nanoparticles predominantly interfere with viral entry and capsid integrity, which reduces collateral damage to host cells and increases selectivity for viral targets. In contrast, copper oxide nanoparticles exhibit potent antiviral properties by inducing oxidative stress that damages viral components; however, this same oxidative stress mechanism also compromises host cell viability, accounting for their lower selectivity [49,54]. These findings are consistent with previous nanomaterial studies describing the higher ROS-mediated cytotoxicity of copper oxide nanoparticles and the relatively favorable biocompatibility of ZnO nanoparticles [55,56].

The antiviral activity against CMV was evaluated based on inhibition of virus-induced cytopathic effects using the crystal violet assay. While this approach is widely used for primary antiviral screening, it does not directly quantify viral replication or infectious viral load. Therefore, the reported IC_50_ values should be interpreted as indicators of CPE inhibition under the experimental conditions rather than definitive measurements of CMV replication inhibition. Future studies employing quantitative PCR, plaque reduction assays, TCID_50_ determination, or viral protein analysis are warranted to confirm the antiviral mechanism.

It should be noted that the in silico docking/MD findings and the in vitro nanoparticle assays address two related but distinct questions: the former evaluates the isolated phytochemical quercetin against modeled/co-folded viral protein targets, while the latter evaluates the intact nanoparticle–extract system against whole-virus infection of cultured cells. The computational data therefore provide a plausible phytochemical-level mechanistic rationale for the extract-capped nanoparticles’ activity rather than direct proof that quercetin mediates the observed cellular antiviral effect; this connection remains to be established experimentally (e.g., via antiviral testing of isolated quercetin). With this scope defined, quercetin was found to be a possible multi-target inhibitor against rotavirus VP5*, CMV UL44, and HPV E6 by integrating explicit-solvent MD simulations with the co-folding model Boltz-2.

Among the flavonoids, quercetin showed the highest predicted affinity across all three targets, and the highest binding likelihood against rotavirus VP5*; for CMV UL44 and HPV E6 the likelihood scores of quercetin and kaempferol were comparable (0.67 vs 0.68 and 0.57 vs 0.63, respectively). According to the MD simulations, the CMV and HPV systems stabilized at 0.41 nm and 0.58 nm after 70 ns, respectively, whereas the rotavirus VP5* complex remained remarkably stable (RMSD ~0.2 nm). Global unfolding events were not seen in RoG or SASA. In line with functional dynamics, the observed RMSF analysis revealed localized flexibility at terminal regions and loop motifs. Detailed analyses of the molecular dynamics trajectories, including the time evolution of protein–ligand interaction energies and hydrogen-bond occupancy, are provided in the Supplementary Figures S1 and S2. Supplementary Figure S1A–C demonstrates that the stability of all complexes was primarily maintained through consistent Lennard-Jones (van der Waals) interactions, while electrostatic contributions exhibited dynamic fluctuations throughout the simulations. In particular, transient increases in Coul-SR interactions were associated with periods of enhanced total binding affinity. Furthermore, Supplementary Figure S2A–C reveal persistent hydrogen-bond formation between quercetin and the target proteins, despite temporal fluctuations in bond numbers. Collectively, these findings support the long-term stability and favorable binding behavior of quercetin within the active sites of CMV UL44, HPV E6, and rotavirus VP5* proteins.

Furthermore, the utilization of green synthesis methods, such as the extraction from *Moringa oleifera* employed in the present study, supports current trends toward sustainable and biocompatible nanomedicine development, which is critical for future clinical translation. The physicochemical characterization performed in this study confirmed the successful synthesis and structural properties of the nanoparticles; however, additional colloidal characterization and stability assessment in biological media are required to fully understand nanoparticle behavior under physiological conditions [57]. Future studies should include DLS, zeta potential, and nanoparticle stability/dissolution profiling in complete cell-culture medium over the assay time-course, since agglomeration or ion release under these conditions directly affects the true exposure dose and cannot be inferred from characterization performed in water alone.

## 5. Conclusions

This study clearly shows that green-synthesized zinc oxide (ZnO-NPs) and copper oxide nanoparticles (CuO-NPs) prepared using *Moringa oleifera* extract exhibit measurable preliminary in vitro antiviral activity against human papillomavirus (HPV), rotavirus, and human cytomegalovirus (HCMV) using a crystal violet-based cytopathic effect (CPE) inhibition assay. ZnO nanoparticles showed superior antiviral performance, lower cytotoxicity, and higher selectivity indices than CuO-NPs, indicating a broader in vitro selectivity window under the experimental conditions employed. In parallel, quercetin, the major flavonoid constituent of *Moringa oleifera*, showed the highest predicted affinity across all three viral targets and stable dynamical behaviour throughout the simulations. The rotavirus VP5* complex showed exceptional conformational stability (RMSD ≈ 0.2 nm), while the CMV UL44 and HPV E6 complexes stabilized after 70 ns and maintained interaction with the protein. These in silico findings provide a strong rationale for validation of quercetin as a multi-target antiviral agent, particularly against rotavirus, cytomegalovirus, and human papillomavirus. Future work should prioritize in vitro binding assays and cell-based antiviral testing.

Also, these findings indicate that ZnO nanoparticles had a wider in vitro selectivity window than CuO nanoparticles under the tested experimental conditions. These findings align with previous studies and further validate the safer antiviral profile of ZnO nanoparticles compared to CuO nanoparticles. These features make ZnO nanoparticles good choices for antiviral uses, as they work well and are safe for cells. However, further virus-specific, mechanistic, and in vivo studies are required before therapeutic application can be proposed.

Overall, this study underscores the potential of green-synthesized ZnO nanoparticles as a sustainable, effective, and safe antiviral agent, warranting further investigation and optimization for therapeutic and prophylactic applications. The promising outcomes suggest that ZnO nanoparticles could play a pivotal role in the development of novel antiviral strategies targeting a wide range of viral infections, making them a viable alternative to conventional antiviral treatments.

### Overall Significance

This appears to be among the first studies to compare quercetin binding across an entry protein, a replication processivity factor and an oncoprotein within a single computational workflow. The finding that one natural flavonoid can stably engage three unrelated viral proteins with moderate to high predicted affinity is consistent with the concept of polypharmacology in natural products. Quercetin has a well-characterized human safety profile; however, its oral bioavailability remains low and variable due to limited solubility, extensive metabolism, and rapid elimination. Nevertheless, quercetin represents a promising lead for therapeutic repurposing and a valuable scaffold for the development of synthetic analogues with improved pharmacokinetic properties.

## Figures and Tables

**Table 1:** Assessment of the cytotoxicity and antiviral activity of ZnO and CuO nanoparticles against human papillomavirus under in vitro assay conditions. BHK-21, crystal violet assay (48–72 h, 37 °C, 5% CO_2_, three independent biological experiments).

Virus	Treatment	CC_50_ (μg/mL)	IC_50_ (μg/mL)	SI (CC_50_/IC_50_)
HPV	Standard (Cidofovir)	123.23	0.86	142.46
	ZnO-NPs	311.28	29.16	10.67
	CuO-NPs	84.21	27.67	3.04

Data are presented as mean values from three independent biological experiments, each conducted with three technical replicates. CC_50_ and IC_50_ values were estimated by nonlinear regression analysis of dose–response curves generated from three independent experiments using GraphPad Prism. SI was calculated as CC_50_/IC_50_.

**Table 2:** Assessment of the cytotoxicity and antiviral activity of ZnO and CuO nanoparticles against rotavirus. MDCK cells, crystal violet assay (48 h, 37 °C, 5% CO_2_, three independent biological experiments).

Virus	Treatment	CC_50_ (μg/mL)	IC_50_ (μg/mL)	SI (CC_50_/IC_50_)
Rotavirus	Standard (Ribavirin)	351.85	29.04	12.11
	ZnO-NPs	798.42	69.43	11.49
	CuO-NPs	133.38	25.41	5.24

Data are presented as mean values from three independent biological experiments, each conducted with three technical replicates. Assays were done in MDCK cells using the crystal violet method. CC_50_ and IC_50_ values were estimated by nonlinear regression analysis of dose–response curves generated from three independent experiments using GraphPad Prism. SI was calculated as CC_50_/IC_50_.

**Table 3:** Assessment of the cytotoxic and antiviral inhibitory effects of ZnO and CuO nanoparticles against cytomegalovirus under in vitro assay conditions. MDCK cells, crystal violet assay (72 h, 37 °C, 5% CO_2_, three independent biological experiments).

Virus	Treatment	CC_50_ (μg/mL)	IC_50_ (μg/mL)	SI (CC_50_/IC_50_)
CMV	Standard (Ganciclovir)	8.34	0.78	10.56
	ZnO-NPs	233.90	24.14	10.57
	CuO-NPs	37.09	27.38	1.35

Data are presented as mean values from three independent biological experiments, each conducted with three technical replicates. Assays were done in MDCK cells using the crystal violet method. CC_50_ and IC_50_ values were estimated by nonlinear regression analysis of dose–response curves generated from three independent experiments using GraphPad Prism. SI was calculated as CC_50_/IC_50_.

**Table 4:** Predicted pIC_50_ values (−log_10_ IC_50_) and binding likelihood scores for selected phytochemicals against viral proteins. Values in parentheses represent the calculated binding likelihood.

	Quercetin	Chlorogenic Acid	Kaempferol	Rutin
Rotavirus VP5*	7.30 (0.67)	6.33 (0.25)	6.88 (0.59)	6.93 (0.50)
HPVE6 Oncoprotein	6.55 (0.57)	6.01 (0.44)	6.20 (0.63)	6.48 (0.49)
CMV UL44	7.40 (0.67)	7.02 (0.44)	6.45 (0.68)	7.00 (0.65)

Values in Table 4 are expressed as predicted pIC_50_ scores (−log_10_ IC_50_) derived from the Boltz-2 co-folding model and are used only for relative computational ranking of ligand–protein interactions, while values in parentheses indicate the corresponding binding likelihood scores; higher binding likelihood values reflect stronger predicted affinity and stability of ligand–protein interactions.

## Data Availability

The original contributions presented in this study are included in the article/Supplementary Material. Further inquiries can be directed to the corresponding authors.

## Supplementary Materials

The following supporting information can be downloaded at: https://www.mdpi.com/article/10.3390/nano16171067/s1. Supplementary data associated with this article include detailed molecular dynamics analyses of the studied protein–ligand complexes, including time-resolved interaction energy profiles and hydrogen-bond evolution throughout the simulation trajectories (Supplementary Figures S1 and S2). These data further support the stability and binding behavior of quercetin against CMV UL44, HPV E6, and rotavirus VP5* targets. Figure S1. Time evolution of protein–ligand interaction energies for the three target proteins: (A) CMV UL44 processivity factor (PDB 1YYP), (B) HPV E6 oncoprotein (PDB 4GIZ), (C) rotavirus VP5* (PDB 1SLQ). Figure S2. Time evolution of protein–ligand hydrogen-bond numbers for the three target proteins: (A) CMV UL44 processivity factor (PDB 1YYP), (B) HPV E6 oncoprotein (PDB 4GIZ), (C) rotavirus VP5* (PDB 1SLQ).

## Abbreviations

HPV	Human Papillomavirus
HCMV	Human Cytomegalovirus
ROTA	Rotavirus
ZnO-NPs	Zinc Oxide Nanoparticles
CuO-NPs	Copper Oxide Nanoparticles
UV–Vis	Ultraviolet–Visible Spectroscopy
XRD	X-ray Diffraction
FTIR	Fourier-Transform Infrared Spectroscopy
TEM	Transmission Electron Microscopy
ROS	Reactive Oxygen Species
DMEM	Dulbecco’s Modified Eagle Medium
FBS	Fetal Bovine Serum
PBS	Phosphate-Buffered Saline
MDCK	Madin–Darby Canine Kidney Cells
BHK-21	Baby Hamster Kidney-21 Cells
MOI	Multiplicity of Infection
CPE	Cytopathic Effect
CC_50_	50% Cytotoxic Concentration
IC_50_	50% Inhibitory Concentration
SI	Selectivity Index
RMSF	Root Mean Square Fluctuation
RMSD	Root Mean Square Deviation
SASA	Solvent Accessible Surface Area
