# Supplementary material for: Antiviral Potential of Green-Synthesized Zinc Oxide and Copper Oxide Nanoparticles: Integrated In Vitro and In Silico Study
Source: Nanomaterials (Basel). 2026 Aug 27;16(17):1067. doi: 10.3390/nano16171067 (PMC13567136; doi:10.3390/nano16171067)
Supplement: Supplementary file 1 [file nanomaterials-16-01067-s001.zip › nanomaterials-4460613-supplementary.pdf]

<u>**Supplementary Data**</u>

A

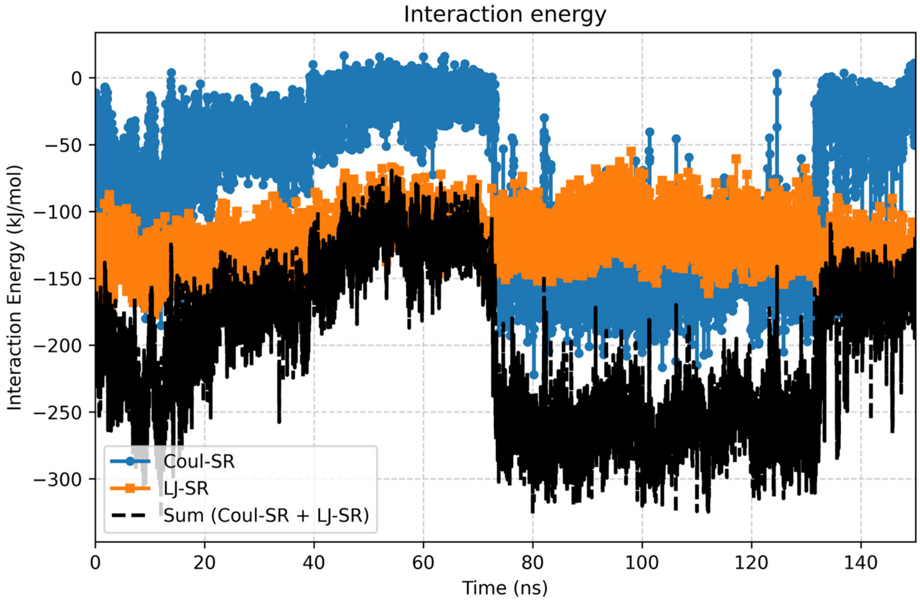

B

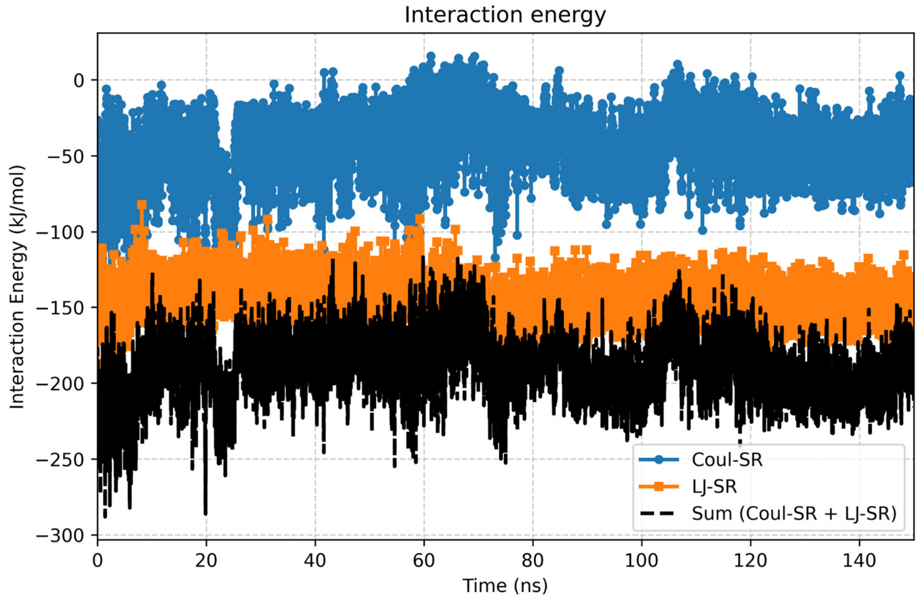

C

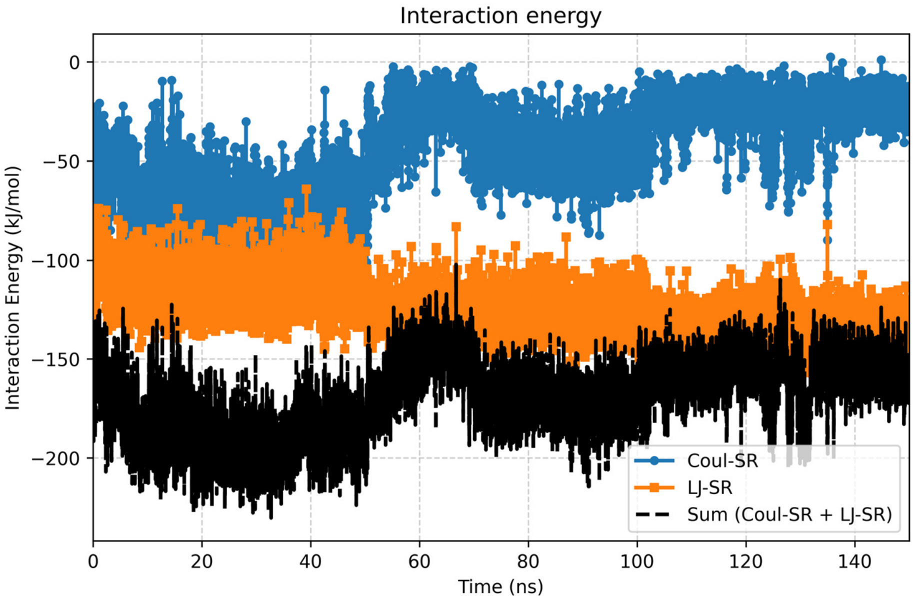

Figure S1. Time evolution of protein–ligand interaction energies for the three target proteins: (**A**) CMV UL44 processivity factor (PDB 1YYP), (**B**) HPV E6 oncoprotein (PDB 4GIZ), (**C**) rotavirus VP5* (PDB 1SLQ).

Short-range protein–ligand interaction energies were decomposed into Lennard-Jones (LJ-SR, van der Waals) and Coulombic (Coul-SR, electrostatic) contributions, and their sum is reported as the total interaction energy. All values are averaged over the 150 ns production trajectory.

Figure S1A shows the interaction energies of quercetin bound to CMV UL44. The complex exhibited a favorable binding profile, with the total interaction energy fluctuating between −320 and −150 kJ/mol. The LJ-SR contribution remained relatively stable (mean ≈ −120 kJ/mol), whereas a marked transition was observed between 75 and 130 ns. During this period the total energy reached its minimum of approximately −320 kJ/mol, driven by a sharp increase in Coul-SR attraction. This pattern indicates that hydrophobic contact provides a constant baseline for stability, while binding strength is periodically maximized by the formation of transient polar interactions, principally hydrogen bonds.

For the HPV E6 oncoprotein (Figure S1B), the complex demonstrated consistent energetic stability throughout the simulation, with total interaction energies predominantly fluctuating between −250 and −175 kJ/mol. Binding was primarily anchored by van der Waals interactions, which maintained a stable profile at approximately −130 kJ/mol, consistent with a well-fitted hydrophobic contact within the binding pocket. The electrostatic component was generally favorable (mean ≈ −50 kJ/mol) but showed greater volatility, with transient excursions toward zero indicating a dynamic polar environment. These results indicate that the complex is governed by a robust van der Waals baseline, with electrostatic fluctuations reflecting the periodic formation and breaking of localized polar contacts.

The rotavirus VP5* complex (Figure S1C) likewise remained energetically stable, with total interaction energies fluctuating between −225 and −130 kJ/mol. Binding was predominantly driven by van der Waals forces, which maintained a highly consistent and favorable baseline of approximately −120 kJ/mol,

indicating strong hydrophobic complementarity and structural fit within the binding site. The electrostatic component exhibited higher volatility, ranging from 0 to −75 kJ/mol. A slight weakening in total binding energy was observed between 50 and 75 ns, coinciding with a reduction in Coul-SR and likely reflecting a transient rearrangement of the hydrogen-bonding network. Overall, the energetic landscape describes a stable complex in which consistent hydrophobic anchoring is supplemented by intermittent electrostatic interactions.

A

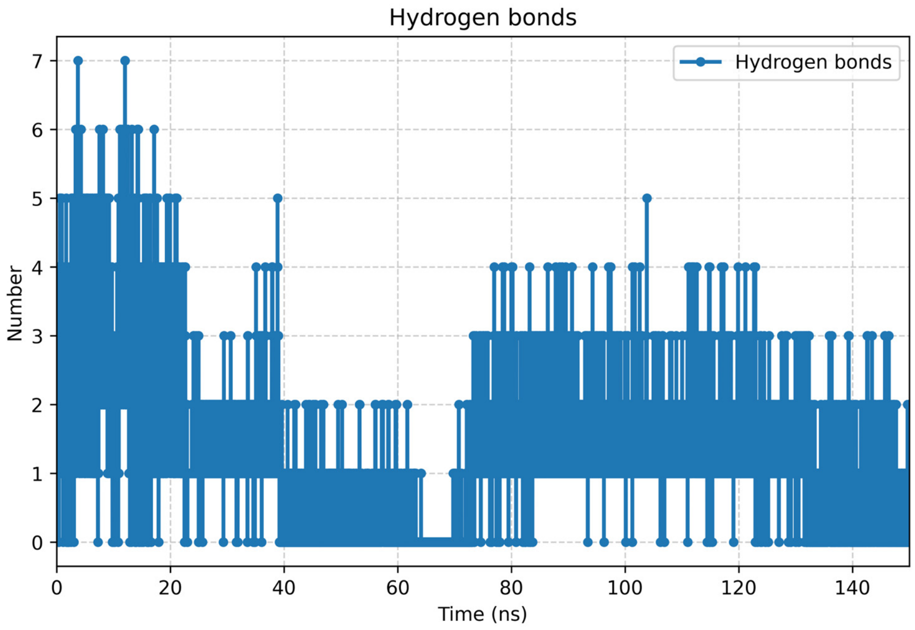

B

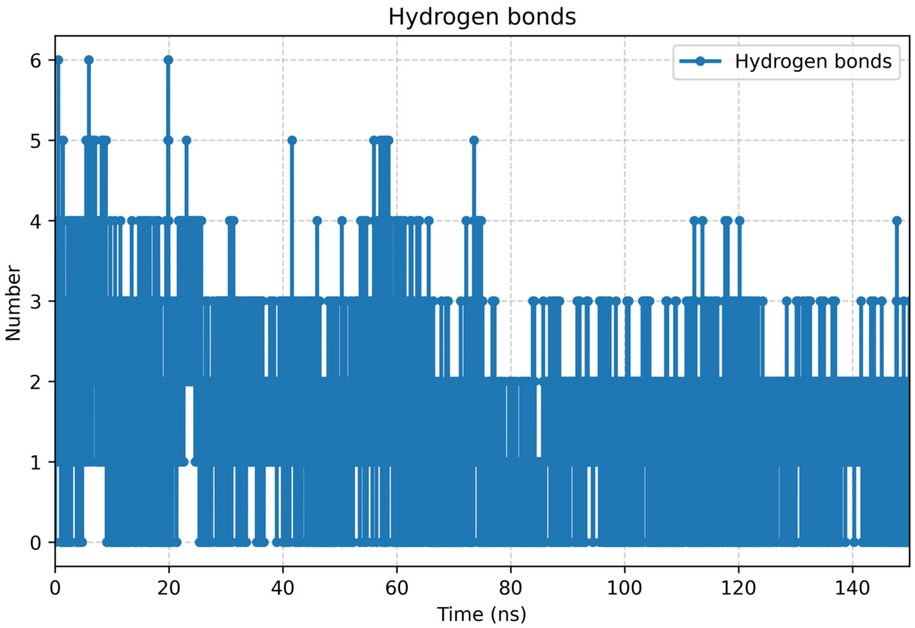

C

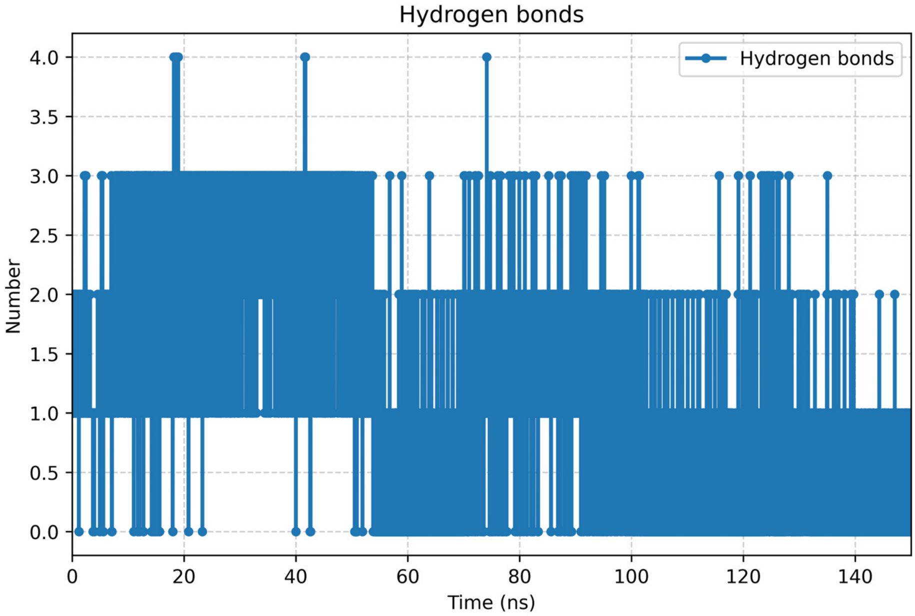

Figure S2. Time evolution of protein–ligand hydrogen-bond numbers for the three target proteins: (**A**) CMV UL44 processivity factor (PDB 1YYP), (**B**) HPV E6 oncoprotein (PDB 4GIZ), (**C**) rotavirus VP5* (PDB 1SLQ).

The hydrogen-bonding profile of the CMV UL44–quercetin complex (Figure S2A) revealed a dynamic interaction landscape with pronounced fluctuations over the 150 ns simulation. During the initial phase (0–20 ns), the complex was stabilized by a high density of hydrogen bonds, peaking at seven concurrent interactions. This was followed by a period of relative instability (20–70 ns), during which the number of hydrogen bonds frequently fell to zero, indicating a conformational rearrangement or a transient weakening of the polar network. From 70 ns onward, hydrogen bonding recovered and stabilized at between one and four bonds for the remainder of the

trajectory. These fluctuations parallel the variations in electrostatic interaction energy shown in Figure S1A, indicating that binding is periodically reinforced by the formation of specific hydrogen-bonded networks.

The HPV E6–quercetin complex (Figure S2B) showed a highly dynamic but persistent polar network. Throughout the simulation the system maintained between one and four concurrent hydrogen bonds. The initial 25 ns exhibited greater volatility, with transient peaks of up to six hydrogen bonds, indicating an actively rearranging binding pocket. Thereafter the interaction pattern remained consistent, generally oscillating between one and three bonds with occasional spikes. The continuous presence of at least one hydrogen bond for most of the trajectory indicates that these polar contacts anchor the ligand and confer specificity despite the conformational flexibility of the complex.

The rotavirus VP5*–quercetin complex (Figure S2C) maintained a consistent hydrogen-bonding network throughout the simulation, predominantly oscillating between one and three concurrent bonds. A distinct transition in bonding density was observed: during the first 55 ns the complex exhibited more robust polar interactions, frequently reaching three to four concurrent hydrogen bonds, whereas from 55 ns to the end of the trajectory the network became more dynamic, with bond numbers fluctuating mainly between one and two. Despite this reduction in bond density, hydrogen bonds were present for the majority of the trajectory, indicating that polar interactions contribute substantially to long-term anchoring of the ligand within the binding pocket.
